# Fasting in the ureotelic Lake Magadi tilapia, *Alcolapia grahami*, does not reduce its high metabolic demand, increasing its vulnerability to siltation events

**DOI:** 10.1093/conphys/coz060

**Published:** 2019-10-31

**Authors:** Gudrun De Boeck, Chris M Wood, Kevin V Brix, Amit K Sinha, Victoria Matey, Ora E Johannsson, Adalto Bianchini, Lucas F Bianchini, John N Maina, Geraldine D Kavembe, Michael B Papah, Mosiany L Kisipan, Rodi O Ojoo

**Affiliations:** 1 SPHERE, Department of Biology, Groenenborgerlaan 171, University of Antwerp, Antwerp B-2020, Belgium; 2 Department of Biology, McMaster University, 1280 Main St W, Hamilton, ON L8S 4K1, Canada; 3 Department of Zoology, University of British Columbia, 6270 Univ Blvd, Vancouver, BC V6T 1Z4, Canada; 4 Rosenstiel School of Marine and Atmospheric Sciences, University of Miami, 4600 Rickenbacker Causeway, Miami, FL 33149, USA; 5 EcoTox, 3211 19th Terrace, Miami, FL 33145, USA; 6 Department of Aquaculture and Fisheries, University of Arkansas, 1200 North Univ Dr, Pine Bluff, AR 71601, USA; 7 Department of Biology, San Diego State University, 5500 Campanile Dr., San Diego, CA 92182, USA; 8 Instituto de Ciências Biológicas, Universidade Federal do Rio Grande, Ave Italia Km 8, Rio Grande, RS 96203-900, Brazil; 9 Department of Zoology, University of Johannesburg, PO Box 524 Auckland Park, Johannesburg 2006, South Africa; 10 School of Dryland Agriculture Science and Technology, South Eastern Kenya University, PO Box 170, Kitui 90200, Kenya; 11 Department of Animal and Food Sciences, University of Delaware, 531 S. College Ave., Newark, DE 19716, USA; 12 Department of Veterinary Anatomy and Physiology, Egerton University, Njoro Campus PO Box 536, Egerton 20115, Kenya; 13 Department of Veterinary Anatomy and Physiology, University of Nairobi, Riverside Drive, Chiromo Campus, PO Box 30197-00100, Nairobi 30197, Kenya

**Keywords:** Alkaline soda lake, starvation, metabolism, respiration, nitrogen, urea-N

## Abstract

Lake Magadi, Kenya, is one of the most extreme aquatic environments on Earth (pH~10, anoxic to hyperoxic, high temperatures). Recently, increased water demand and siltation have threatened the viable hot springs near the margins of the lake where *Alcolapia grahami*, the only fish surviving in the lake, live. These Lake Magadi tilapia largely depend on nitrogen-rich cyanobacteria for food and are 100% ureotelic. Their exceptionally high aerobic metabolic rate, together with their emaciated appearance, suggests that they are energy-limited. Therefore, we hypothesized that during food deprivation, Magadi tilapia would economize their energy expenditure and reduce metabolic rate, aerobic performance and urea-N excretion. Surprisingly, during a 5-day fasting period, routine metabolic rates increased and swimming performance (critical swimming speed) was not affected. Urea-N excretion remained stable despite the lack of their N-rich food source. Their nitrogen use switched to endogenous sources as liver and muscle protein levels decreased after a 5-day fast, indicating proteolysis. Additionally, fish relied on carbohydrates with lowered muscle glycogen levels, but there were no signs indicating use of lipid stores. Gene expression of gill and gut urea transporters were transiently reduced as were gill rhesus glycoprotein Rhbg and Rhcg-2. The reduction in gill glutamine synthetase expression concomitant with the reduction in Rh glycoprotein gene expression indicates reduced nitrogen/ammonia metabolism, most likely decreased protein synthesis. Additionally, fish showed reduced plasma total CO_2_, osmolality and Na^+^ (but not Cl^−^) levels, possibly related to reduced drinking rates and metabolic acidosis. Our work shows that Lake Magadi tilapia have the capacity to survive short periods of starvation which could occur when siltation linked to flash floods covers their main food source, but their seemingly hardwired high metabolic rates would compromise long-term survival.

## Teaser

Lake Magadi tilapia live near hot water springs in an extremely alkaline lake. They show very high metabolic rates and are 100% ureotelic. During food deprivation, rather than lowering their metabolic activity, they switched to tissue proteolysis, increased their routine O2 consumption, and maintained unchanged swimming performance and urea-N excretion.

## Introduction

**Figure 1:**
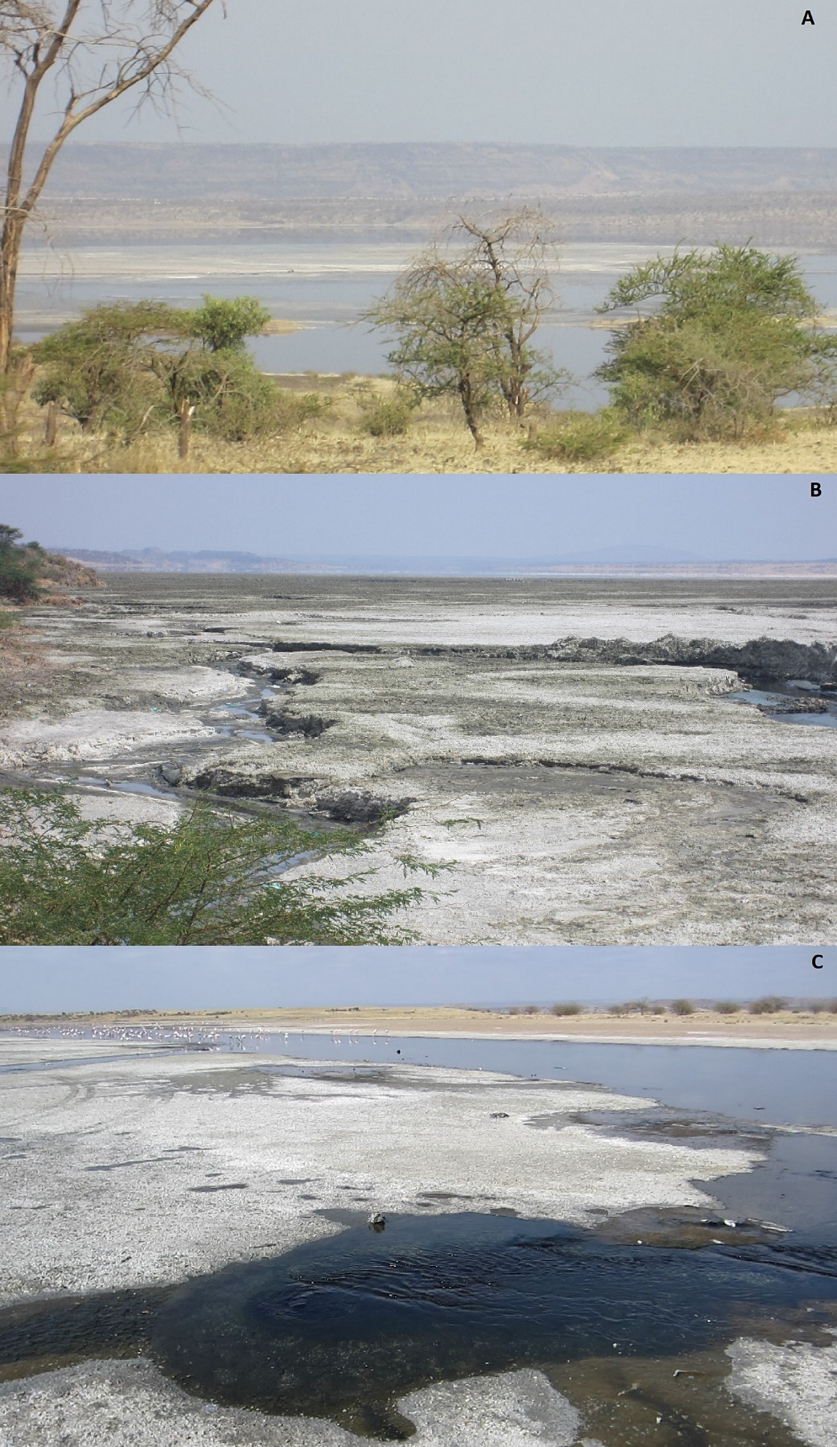
**A**: View of Lake Magadi with the white soda crust partly covered due to heavy rains. **B**: Soda crust as seen from Magadi village. **C**: Upwelling water feeding the lake clearly visible in one of the springs at South West Hot Springs.

East African Rift valley alkaline soda lakes are an unusual and harsh habitat for living organisms. However, even when covered with thick soda crusts, they still contain viable niches in the lake margins, lagoons and the nearby springs that supply the lakes with volcanic groundwater (Fig. 1) (Coe 1966, Kavembe **et al**., 2016). One of these lakes is Lake Magadi in Kenya that has an extremely high pH (up to 10.0), extreme alkalinity (>300 mmol L^−1^), high temperature (>40°C at some sites), high levels of reactive O_2_ species (>8 μmol L^−1^ at some sites during daytime irradiation by high UV light levels), a salinity close to 60% seawater, and large daily fluctuations in O_2_ levels ranging from anoxia or hypoxia to hyperoxia (Coe, 1966; Johansen *et al*., 1975., Narahara *et al*., 1996; Johannsson *et al*., 2014, Wood *et al*., 2016). Nevertheless, it is inhabited by the Lake Magadi tilapia, *Alcolapia grahami* (Boulenger), the only fish species that survives there. Species that survive in extreme conditions have generally evolved special adaptations to do so, and the Lake Magadi tilapia is no exception. They are unique among teleosts in that they have a fully functional ornithine–urea cycle and are 100% ureotelic under all circumstances, an adaptation to the challenges of excreting ammonia into a pH 10 environment (Randall *et al*., 1989, Wood *et al*., 1989, Lindley *et al*., 1999). However, this unique fish is under threat from anthropogenic factors, and in 2006, *A. grahami* was placed on the IUCN Red List of Threatened Species, where it is classified as vulnerable (Bayona and Akinyi, 2006).

The tilapia live near the hot springs surrounding the lake, but the water supply from these sources is now in competition with boreholes that are being sunk around the lake to supply water to the growing local Maasai community and their cattle (Africa Geographic, 2011). Perhaps an even more severe threat is the increase in silt deposits in the lake caused by increased agricultural activity and deforestation, aggravated by road construction with inadequate culverts to drain storm water (Ministry of Environment and Forestry, 2015). Flash floods were reported to have deposited as much as 8000 t of silt after every storm into Lake Magadi (KBC Channel 1, 2015; People Daily, 2018). Such large deposits could cover the rocks encrusted with the thriving cyanobacteria *Arthrospira* sp., which comprise 90% of the Lake Magadi tilapia diet, which is complemented occasionally with copepods and chironomid larvae (Coe, 1966). As the fish often live in isolated springs near the edges of the soda crust, evasion possibilities are limited to absent which could leave them temporarily without access to food. Even under normal circumstances, the fish remain small and often have a very thin appearance, indicating that their energy expenditure might outweigh their energy gain.

**Figure 2:**
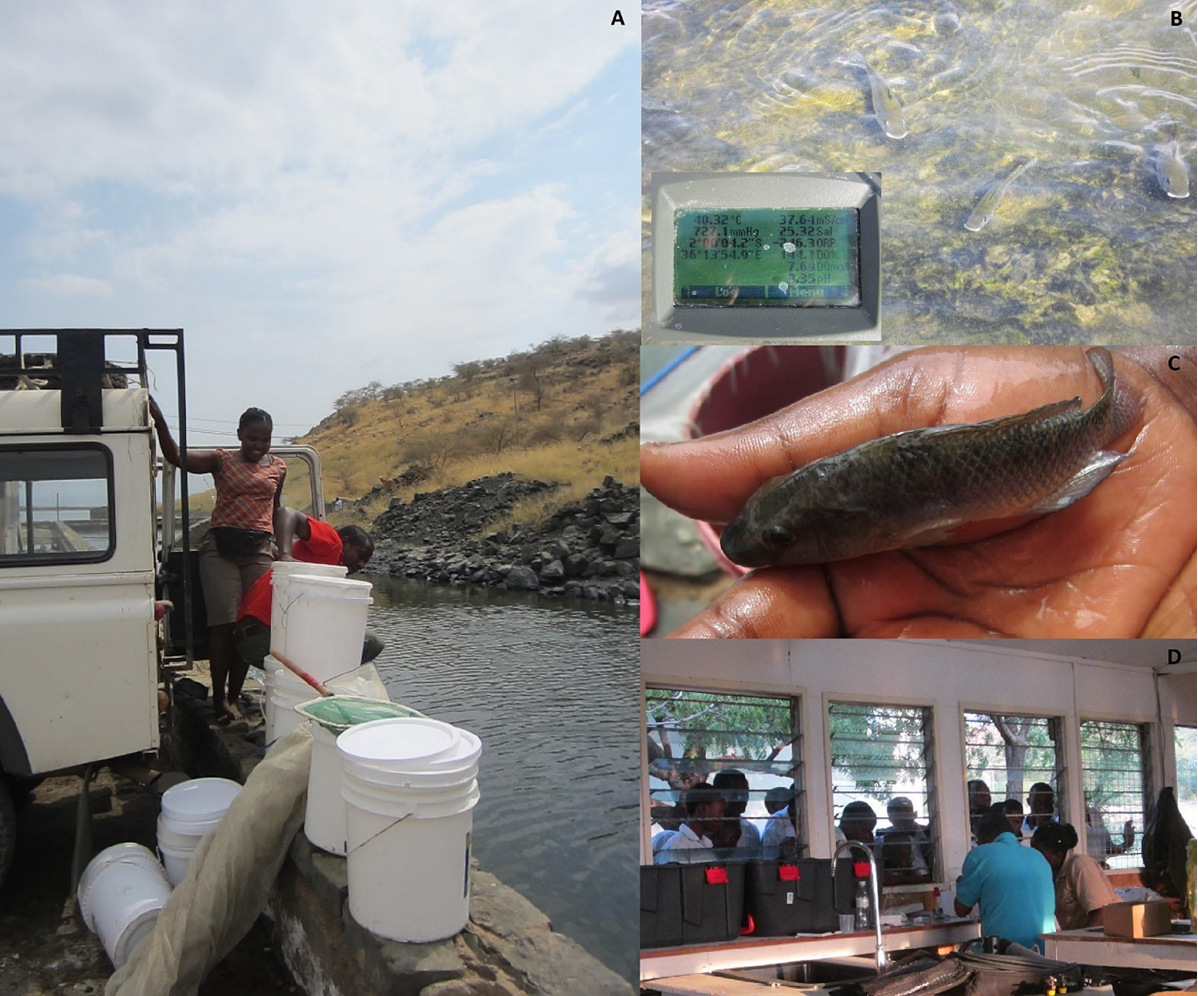
**A**: Seine fishing and water hauling at Fish Springs Lagoon. **B**: Lake Magadi Tilapia in their natural habitat, here at South West Hot Springs. Note the abundant green cyanobacteria, their main food source and the typical white lips displayed by males, as well as the measurements taken at the same time: °T: 40.32°C, DO: 144.1% saturation and pH: 9.35. **C**: Fully grown adult fish. **D**: Working at the Magadi High School chemistry lab ensured continuous interaction with interested students. Note the grey tanks on the left containing the respirometer.

The routine aerobic metabolic rate of the Magadi tilapia is exceptionally high (Franklin *et al*., 1995; Narahara *et al*., 1996, Wood *et al*., 2016). Even when taking the high temperatures into account, Lake Magadi tilapia show metabolic rates that are 25–60% higher than in other tilapia species at similar temperatures (Narahara *et al*., 1996). Indeed, the cost of merely surviving this harsh environment is high and clearly related to the continuous ionoregulatory and acid–base challenges that these fish face, forcing them to excrete nitrogen as urea. Acclimation to dilutions of Lake Magadi water reduced MO_2_ by up to 60%, largely because of reduced acid–base regulatory costs (Wood *et al*., 2002a) which comprise 50–60% of routine metabolism (Wood *et al*., 2002a). By some controversial theories, urea production in the ornithine–urea cycle, through its role in HCO_3_^−^ consumption, is linked to acid–base regulation (Meijer *et al*., 1990; Atkinson, 1992). Regardless, the high metabolic rate may be attributed in part to the fact that urea synthesis is costly (2.5 ATP per urea-N), and Magadi tilapia excrete enormous amounts of urea-N at typical rates of 5000–10 000 μmol urea-N.kg^−1^.h^−1^ (Wood *et al*., 1989). *Alcolapia grahami* endemic to the nearby South West Hot Springs (SWHS, Figs. 1c and 2b) live at temperatures near 41°C and show routine metabolic rates of 88 μmol.g^−1^.h^−1^ which are similar to those of a small mammal (Wood *et al*., 2016). These are three to six times higher than the already exceptionally high values of 15–30 μmol.g^−1^.h^−1^ seen in the more commonly studied Magadi tilapia from Fish Springs Lagoon (FSL; Fig. 2a and c) where the temperatures are between 30 and 35°C (Wood *et al*., 1994, 2002a, 2016; Franklin *et al*., 1995; Narahara *et al*., 1996).

To support these high metabolic rates, Lake Magadi tilapia are voracious feeders, continuously scraping blue-green algae from the rocks (Coe, 1966, Wood *et al*., 1994). Fish were even observed to jump repeatedly out of the water to bite pieces of the cyanobacterial math on a stone wall, resulting in a graze line above the water level (Narahara *et al*., 1996). This was demonstrated by their stomach content containing 90% algae, with the remaining 10% consisting of dipterous larvae and copepods (Coe, 1966). These algae contain about 60% protein and thus are an energy- and nitrogen-rich food source which allows the fish to sustain their high metabolic rates. It is therefore no surprise that Lake Magadi tilapia deteriorate rapidly in captivity (Wood *et al*., 1989, 1994) unless precautions are taken to minimize aggression and provide sufficient food (Narahara *et al*., 1996, Wood *et al*., 2002a). To date, most published studies therefore utilized fish that were caught only hours before experiments took place. In the only experiment comparing the physiology of fed versus fasted fish, mortality was high and only 20% of the fish survived up to the fourth day (Wood *et al*., 1994).

In the present study, a new attempt was made to study fasted Lake Magadi tilapia taking the necessary precautions as described by Narahara and co-workers (1996) who advised to keep densities low enough to avoid aggression and to shield the holding tanks from light. To avoid feeding on extraneous sources, extreme caution was taken to keep the tanks devoid of waste and algae, and dead fish were removed immediately. Our aims were to study metabolic rates and utilization of on-board fuels. We hypothesized that the fish would economize energy use and thus lower their routine MO_2_ in the absence of food. A second hypothesis was that urea-N effluxes would be reduced when the fish were deprived of the N-rich cyanobacteria. Therefore, urea-N fluxes and plasma urea, as well as gill, gut and kidney urea transporter mRNA expression were assessed, complemented by mRNA expression of ammonia transporters and glutamine synthetase as a proxy for protein metabolism. Overall, we expected fish to be energy-limited and perform less well, and this was tested by measuring swimming capacity and plasma and tissue metabolites. Finally, gill morphology was examined as our earlier work with an Amazonian cichlid (*Astronotus ocellatus*) showed clear interactions between gill morphology and physiology, where fasted fish had more mitochondria-rich cells with larger crypts, indicating the increased importance of the branchial uptake route when feeding is limited (De Boeck *et al*., 2013).

## Material and methods

All research was performed under a research and ethics clearance permit (NCST/RR1/12/1/MAS/99) from the National Commission for Science, Technology and Innovation (NACOSTI Kenya), and with the permission of the Magadi Soda Foundation. All foreign researchers were licensed by NCST and formally appointed as visiting researchers at the University of Nairobi. Collections were made under permission from the Department of Fisheries, Ministry of Livestock and Fisheries (Kenya).

### Experimental animals

All Magadi tilapia, *A. grahami*, were collected by beach seine from Fish Spring Lagoons (FSL) at Lake Magadi, Kenya (GPS coordinates = 1°53′30.2″S, 36°18′09.9″E) in July and August 2013. Weights of fish (*n* = 16) used for the determination of critical swimming speeds were 5.46 ± 0.38 g. Additional fish (*n* = 56) were used for blood and tissue sampling with an average weight of 6.61 ± 0.23 g. Due to the size of the respirometers, fish used for respirometry (*n* = 16) were smaller and averaged 2.44 ± 0.15 g. All were transported together with water from the site to the nearby lab set up in a classroom of Magadi Secondary School, where they were kept outdoors in small groups (<30) in 100-L tanks equipped with standard aquarium heaters in well-aerated FSL water (T°: 30–32°C, pH 9.80, conductivity 2.45 S/cm, salinity 16.6 ppt). Thus, temperature was slightly lower than in the field which showed diurnal temperature variations of 33–36°C and a pH of 9.75 during that period (Wood *et al*., 2016). Therefore, we might have slightly underestimated metabolic rates. Tanks were shielded from the light by a lid, and 75% of the water was renewed daily using fresh FSL water to ensure good water quality.

For control measurements, all fish were allowed to recover from capture stress for a few hours and were always sampled within 24 h after capture. For the fasting period, fish were maintained as above and care was taken that tanks were clean at all times, any waste was syphoned off three times a day, and any fish that were dead or dying were removed. After the first 24 h, no more mortality occurred, guts were cleared of all food, and waste accumulation was minimal.

### Swimming performance and respirometry

Fish were placed in four small-scale (3.9 L) Blazka-style swimming tunnels, 1 h before measurement (one fish per respirometer). Water speed was set at 10 cm/s which allowed the fish to orient themselves against the water current and swim gently. Swimming respirometers were submerged in individual water tanks containing fresh, well-aerated FSL water, and an aquarium heater maintained the temperature at 30–32°C. A small circulation pump was used to circulate this well-aerated water into the swimming chambers during the trials.

Water velocity was changed in increments of 10 cm/s at 20-min intervals, until fish became fatigued. Fish were considered fatigued once they drifted against the rear screen and would not swim for 30 s after the water velocity was temporarily lowered and then returned to the speed at which exhaustion had occurred. *U*_crit_ was calculated according to the equation given by Brett (1964):

$$ {U}_{\mathrm{crit}}={U}_i+\left[{U}_{ii}\left(\frac{T_i}{T_{ii}}\right)\right] $$

where *U_i_* is the penultimate swim velocity at exhaustion, *U_ii_* is the velocity increment (10 cm/s), *T_i_* is the time elapsed at fatigue velocity, and *T_ii_* is the interval time (20 min). The absolute values (cm/s) were converted to relative swimming speeds in BL/s by dividing the absolute values by the fish fork length.

As the resolution for oxygen consumption rates (*M*_O2_) and urea-nitrogen excretion rates (*J*_urea-N_) during the swimming protocol was not high enough, separate respiration trials were performed in 530-mL amber glass bottles (‘Tusker chambers’, Wood *et al*., 1989) fitted with aeration and submerged in a water bath at 30–32°C. Eight chambers were filled with clean well aerated water after which fish were allowed to settle for 1 h in the chamber with continuous aeration. At the start of oxygen consumption measurements, the aeration was removed, and an initial water sample was collected from each chamber. Oxygen was measured using an electrode (WTW CellOx 325, Germany) set at the appropriate salinity, and subsequently, the chambers were sealed for 1 h which was sufficient to allow oxygen levels to drop to approximately 2 mg.L^−1^. At that point, oxygen level was again determined after which aeration was restored. After 5 h, a final water sample was collected to measure urea-N excretion by fish. Urea levels were determined with the diacetyl monoxime method (Price and Harrison, 1987) and multiplied by 2 to obtain urea-N.

### Blood and tissue sampling and analysis

For blood and tissue sampling, fish were sedated in FSL water with clove oil (200 mg/L). Blood samples were immediately taken with a heparinized 100-μL Hamilton syringe (1000 U/mL in Cortland saline). In one subset of fish, blood glucose and lactate were immediately determined using an Accu-Check Compact Plus Meter (Roche Diagnostics, Mannheim, Germany) and a Lactate Pro Meter (ARKRAY Inc., Kyoto, Japan). Other studies have validated the use of such point-of-care portable devices for use in fish as reviewed by Stoot and co-workers (2014). The Lactate Pro Meter was reliable both in relative and absolute values (Brown *et al*. 2008, Serra-Llinares *et al*. 2012), while the Accu-Check tended to underestimate absolute values but was reliable to compare relative values (Cooke *et al*., 2008, Wells and Pankhurst, 1999). The remaining blood was transferred to a heparinized bullet tube, centrifuged for 5 min at 5500 rpm in (NovaTecnica NT800, Sao Paulo, Brazil) and snap-frozen in liquid N_2_ for later determination of plasma urea-N. After cervical section, gill, gut, liver, muscle and kidney were dissected. Liver and muscle were snap-frozen in liquid N_2_ and stored in a −20°C freezer, and gill, gut and kidney transferred to RNAlater (Sigma R0901) and kept at 4°C for later molecular analyses.

In a second subset of fish, blood was immediately centrifuged and plasma frozen as above for later determination of electrolytes and osmolality. Second and third gill arches of these fish were dissected, quickly rinsed and immediately fixed in cold Karnovsky’s fixative (Karnovsky, 1965) and kept cold (4°C) for morphology. Plasma [Na^+^], [Cl^−^] and [K^+^] were analysed using an AVL 9180 Electrolyte Analyzer (AVL, Roche Diagnostics, Belgium), and osmolality using an Advanced™ Micro Osmometer (Model 3300, Advanced Instruments, USA). On a final set of remaining fish from the 5-day fasted group and some control fish, plasma total CO_2_ was measured in duplicate on 50-μL plasma samples using a Corning model 965 CO_2_ analyser (Lowell, MA, USA).

Frozen liver and muscle samples were later homogenized in four parts of deionized water using a Sonicator (Sonozap, 25 kHz Battery operated ultrasonic processor). They were analysed spectrophotometrically (Elx800 Universal Microplate Reader, Bio-Tek Instruments Inc.) for protein content by Bradford’s method (Bradford, 1976), glycogen content using anthrone reagent (Roe and Dailey, 1966) and lipid content according to Bligh and Dyer (1959). Approximate energy contents of the different energy stores were calculated assuming an energetic content of 17.1 kJ per gram of carbohydrates, 38.9 kJ per gram of lipids and 17.6 kJ per gram of protein (Randall *et al*., 1997).

### Gene expression analysis

After return of the samples (stored in RNAlater) to the University of Antwerp in Antwerp, Belgium, total RNA was isolated from gill, gut and kidney samples using TRIzol (Invitrogen, Merelbeke, Belgium) according to the manufacturer’s instructions. The extracted RNA samples were DNAse-treated to avoid genomic DNA contamination. The quantity of the RNA was evaluated using NanoDrop spectrophotometry (NanoDrop Technologies, Wilmington, DE, USA). The integrity (quality) was checked by QIAxcel Advanced system (Qiagen, Hilden, Germany) and the purity by measuring the OD260/OD280 absorption ratio (>1.95).

For quantitative real-time PCR (qPCR), a starting amount of 1 μg RNA was transcribed into first-strand cDNA using the RevertAid H minus First-Strand cDNA synthesis Kit (Fermentas, Cambridge, UK). Expression of mRNA in the gills, gut and kidney of Magadi tilapia subjected to fasting was compared with that in control fish by qPCR using the specific primers listed in Table 1. The primer sequences were adopted from Wood *et al*. (2013).

**Table 1:** PCR primer sequences and calculated efficiency

Gene	Primer sequence (5′ → 3′)	Efficiency (%)
Rhbg	Forward: TATGGCTTCAGCAGTGTTGG Reverse: TCCAAACGAGATCAGCACAG	120
Rhcg2	Forward: CTGCTGTGCTGGATCTCTGA Reverse: CATGGAGCCACCAGAATCTT	116
Na^+^/K^+^-ATPase	Forward: TTGGAGGCCGTTGAGACTCT Reverse: TGTCAAACCACATGTGAGCC	106
Glutamine synthetase	Forward: TCGCATTCCTCGTAATGTTG Reverse: TCGTTCAGCAAACAGGTGCG	105
Urea transporter	Forward: ATGGCACACCCTGACTTACC Reverse: CCATCCATTTTCCAAACACC	112
ß-Actin	Forward: GCCCATCTACGAGGGTTATG Reverse: GAAGGAGTAGCCACGCTCTG	115

qPCR analyses were performed on an Mx3000P QPCR System (Agilent Technologies, Belgium). Reactions containing 5 μL of 5× diluted cDNA, 10 pmol each of forward and reverse primers, 0.4 μL ROX dye (1:500 dilution) and 10 μL Brilliant II SYBR Green qPCR (Agilent) were performed in a four-step experimental run protocol: a denaturation program (10 min at 95°C), an amplification and quantification program repeated 40 times (30 s at 95°C, 50 s at 55°C, 40 s at 72°C), a melting curve program (55–95°C with a heating rate of 0.10°C/s and a continuous fluorescence measurement) and finally a cooling step. Melt curve analyses of the target genes and reference genes resulted in single products with specific melting temperatures. In addition, ‘no-template’ controls (i.e. with water sample) for each set of genes were also run to ensure no contamination of reagents or primer–dimer formation. Data were extrapolated from standard curves generated by serial dilution of one randomly selected control sample. β-Actin mRNA expression remained stable across treatments and was used for normalization.

### Gill morphology

Gills of a total of 12 fish, 4 controls (fed) and 8 from the experiment (4 fasting for 3 days and 4 fasting for 5 days) were examined by scanning electron microscopy (SEM) and light microscopy (LM). Fixed gills were transported to San Diego State University (SDSU), CA, USA. At SDSU, each gill arch was cut into two equal pieces which contained 10–12 filaments in both anterior and posterior rows for SEM and LM studies. All samples were rinsed in phosphate buffered saline (PBS), postfixed in 1% osmium tetroxide for 1 h and dehydrated in ascending concentrations of ethanol from 30 to 100%. Samples that were visualized using SEM study were critical-point dried, mounted on the stubs, sputter-coated with gold and examined with a Quanta 450 scanning electron microscope (FEI) (Hillsboro, OR, USA) at the accelerating voltage of 3–5 kV. Samples that were examined using LM after dehydration in ethanol were exposed through a graded acetone series to 100% acetone and embedded in EPON epoxy resin (Hexion Inc., Columbus, Ohio). Longitudinal semi-thin sections (1 μm) were made parallel to the long axis of the filaments and were cut with an ultramicrotome (Leica EM microtome, Bannockburn, IL, USA). Then, sections were mounted on glass slides, stained with 0.5% methylene blue and examined in a Nikon Eclipse E200 microscope (Melville, NY, USA) and used for morphometric analysis. The data were based upon 40 measurements per each characteristic (10 randomly selected filaments in 4 fish per condition). The following parameters were measured to estimate the volume of interlamellar cell mass (ILCM): L—basal length of protruding lamellae (determined by SEM), h—ILCM height (determined by LM) and d—distance between two adjacent lamellae (determined by LM). The volume of ILCM was calculated according to Sollid *et al*. (2003) as follows: *V = dhL*.

### Statistics

All data have been presented as mean values ± standard error (SEM). The normality of the data was checked using the Kolmogorov–Smirnov test and data log-transformed as needed. For comparisons among different experimental groups, a one-way analysis of variance (ANOVA) was performed followed by Fisher’s least significant difference (LSD) test. Student’s unpaired two-tailed t-test was used for single comparisons for the swimming performance and respirometry data. The data were analysed using GraphPad Prism 7.00. A probability level of 0.05 was used for rejection of the null hypothesis.

## Results

**Figure 3:**
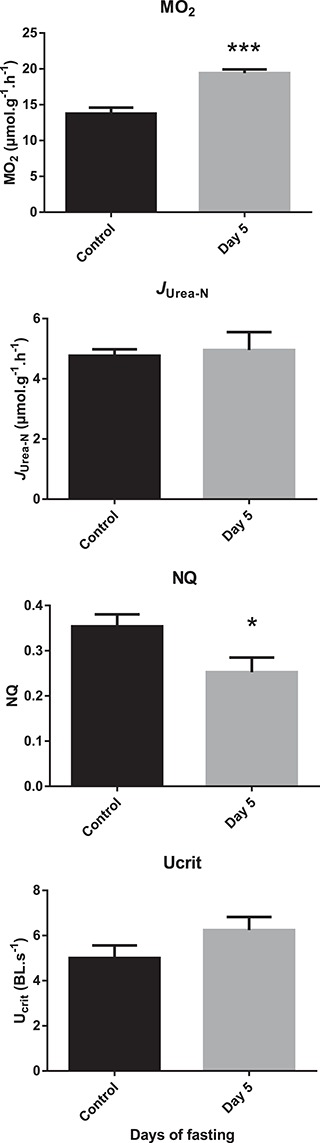
Routine oxygen consumption (MO_2_), urea-N excretion (*J*_urea-N_), nitrogen quotient (NQ) and critical swimming speed (U_crit_) of Magadi tilapia before and after 5 days of fasting. Values are expressed as means and SEM. Asterisks indicate a significant difference between the fasted fish (*n* = 8) and the control fish (*n* = 8) (^*^P < 0.05, ^***^P < 0.001).

Magadi tilapia survived well during the fasting period after an initial typical mortality of around 10–20% during the first night after capture. In general, we did not observe any further mortality or any significant reduction in weight, hepatosomatic index or Fulton condition index (results not shown). Figure 3 shows the typically high routine metabolic rates starting at 13.8 ± 0.8 μmol.g^−1^.h^−1^ in control fish, which significantly increased to 19.4 ± 0.5 μmol.g^−1^.h^−1^ when fish were fasted for 5 days. Despite withdrawal from their N-rich food source, urea-N excretion rates (*J*_urea-N_) did not change and remained just below 5 μmol.g^−1^.h^−1^. As a consequence of the increased metabolic rate, the nitrogen excretion to oxygen uptake ratio (NQ) decreased with fasting. Also, swimming performance, measured as critical swimming speed *U*_crit_, tended to increase, albeit not significantly.

**Figure 4:**
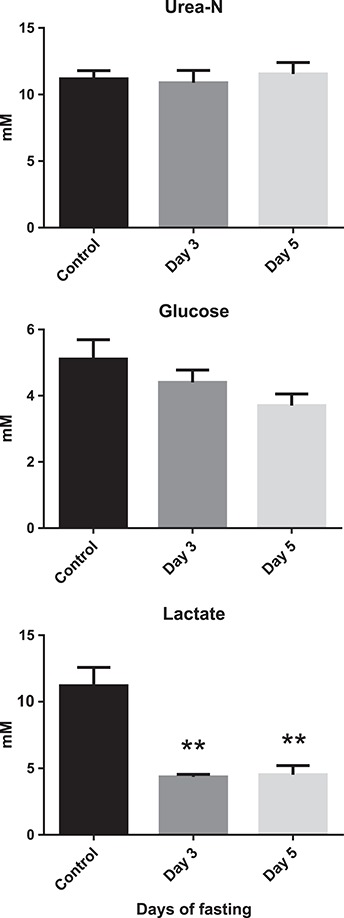
Plasma metabolites: urea-N, glucose and lactate of Magadi tilapia before and during fasting. Values are expressed as means and SEM. Asterisks indicate a significant difference between the fasted fish (*n* = 8) and the control fish (*n* = 8) (^**^P < 0.01).

**Figure 5:**
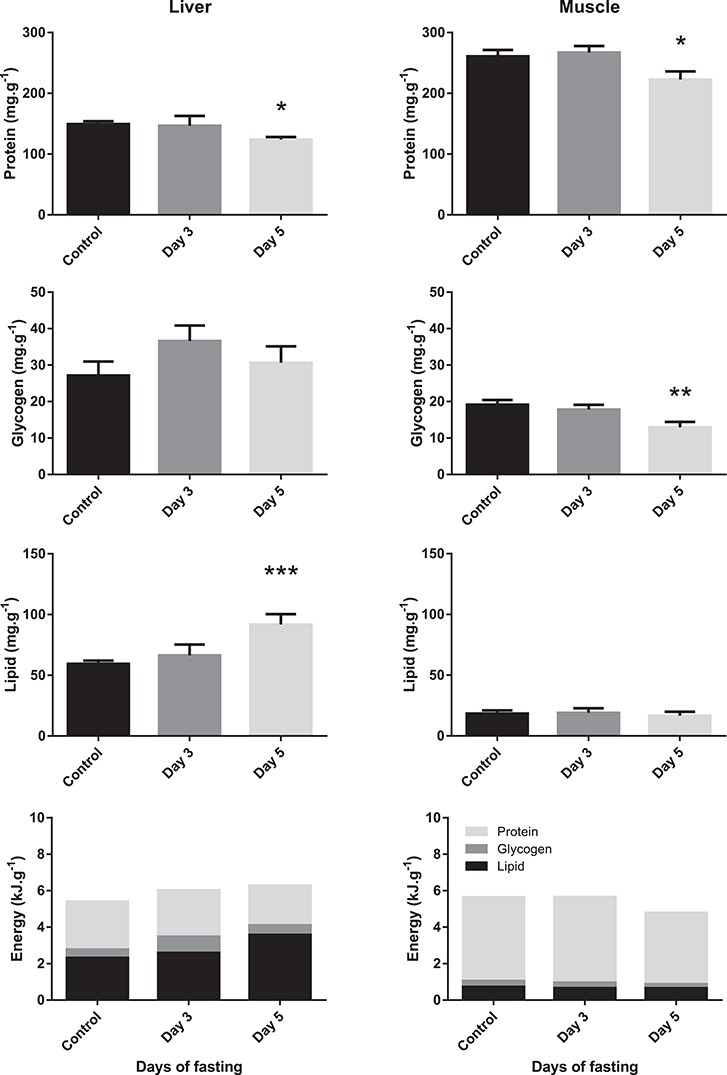
Liver and muscle energy stores (protein, glycogen and lipid) and energetic content (kJ) of Magadi tilapia on a per gram wet weight basis before and during fasting (light grey: protein; dark grey: glycogen; black: lipids). Values are expressed as means and SEM. Asterisks indicate a significant difference between the fasted fish (*n* = 8) and the control fish (*n* = 8) (^*^P < 0.05, ^**^P < 0.01).

Plasma urea-N remained very stable throughout the fasting period (Fig. 4). A non-significant trend of decreasing plasma glucose levels could be seen, but plasma lactate showed a substantial drop from 11 to 4.5 mM which was complete by 3 days of fasting. Also liver and muscle protein levels were reduced by approximately 15% after 5 days of fasting (Fig. 5). While liver glycogen levels remained stable, muscle glycogen decreased by approximately 30%. In contrast, liver lipid concentrations increased after 5 days of fasting, while no changes were seen in muscle lipid levels. When calculating energy content of the tissues (protein: 17.6 kJ.g^−1^, carbohydrates: 17.1 kJ.g^−1^ and lipids: 38.9 kJ.g^−1^), lipid was the main energy source present in liver tissue while protein was the main source in muscle tissue.

**Figure 6:**
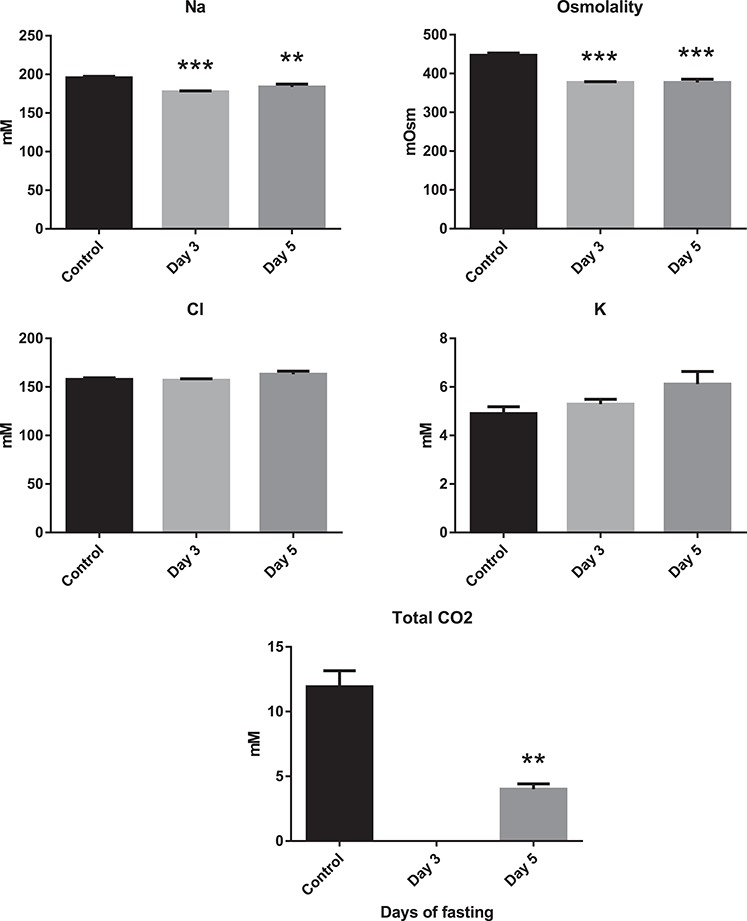
Plasma ions, osmolality and total CO_2_ of Magadi tilapia before and during fasting. Values are expressed as means and SEM. Asterisks indicate a significant difference between the fasted fish (*n* = 8) and the control fish (*n* = 8) (^**^P < 0.01, ^***^P < 0.001).

Plasma Na^+^ and osmolality quickly decreased by Day 3 of fasting (Fig. 6). Plasma Na^+^ dropped from 195 to about 180 mM by Day 3 of fasting, while osmolality dropped from 447 to 377 mOsm, deviating further from Fish Springs Lagoon water, which had Na^+^ levels of 392 mM and a total osmolality of 513 mOsm during our sampling period (Wood *et al*., 2016). Plasma Cl^−^ and K^+^ remained unchanged. Total CO_2_ in plasma was only measured before and after 5 days of fasting but decreased by 66% from 11.9 ± 1.2 to 4.0 ± 0.4 mM, again substantially lower than the 165 mM measured in Lake Magadi water (Wood *et al*., 2016).

Gene expression results of key transporters in the gills show clear changes in the mRNA expression of rhesus glycoproteins (Table 2). Rhbg mRNA expression decreased by about 85% at Day 3 of fasting with some recovery thereafter, while the expression of Rhcg-2 exhibited a similar reduction and remained depressed on both Day 3 and Day 5 of fasting. Also, the urea transporter showed a 35% transient reduction at Day 3 of fasting. No change was seen in Na^+^/K^+^-ATPase mRNA expression. Glutamine synthetase showed a 50% reduction in mRNA expression rates at both Days 3 and 5 of fasting. In the gut (Table 2), there were no significant changes in either of the Rh glycoproteins mRNA expression levels, but there was a 75% drop in urea transporter mRNA expression on Day 3 of fasting. The 30% drop in glutamine synthetase mRNA expression was not significant. No significant differences were seen in the expression of any of these genes in the kidney either.

**Table 2:** Relative expression of mRNA of rhesus glycoproteins Rhbg and Rhcg-2, urea transporter (UT), Na^+^/K^+^-ATPase (NKA) and glutamine synthetase (GS) in the gills of Magadi tilapia before and during fasting; values are expressed as means and SEM; asterisks indicate a significant difference between the fasted fish (*n* = 6–9) and the control fish (*n* = 7–8) (*P < 0.05, **P < 0.01)

		Rhbg	Rhcg-2	UT	NKA	CS
**Gill**	**Control**	1.229 ± 0.261	1.014 ± 0.223	0.090 ± 0.008	0.107 ± 0.011	0.500 ± 0.056
	**Day 3**	0.174 ± 0.045^**^	0.163 ± 0.065^**^	0.056 ± 0.005^**^	0.134 ± 0.033	0.248 ± 0.029^**^
	**Day 5**	0.561 ± 0.208	0.135 ± 0.04^**^	0.082 ± 0.010	0.089 ± 0.013	0.274 ± 0.049^**^
						
**Intestine**	**Control**	0.004 ± 0.001	0.002 ± 0.001	0.260 ± 0.068	1.585 ± 0.248	23.275 ± 2.440
	**Day 3**	0.003 ± 0.001	0.005 ± 0.002	0.066 ± 0.011^*^	2.291 ± 0.287	15.757 ± 2.019
	**Day 5**	0.003 ± 0.001	0.007 ± 0.004	0.126 ± 0.053	1.532 ± 0.219	18.392 ± 2.956
						
**Kidney**	**Control**	0.078 ± 0.031	0.513 ± 0.221	1.366 ± 0.228	1.263 ± 0.169	1.528 ± 0.233
	**Day 3**	0.060 ± 0.048	0.150 ± 0.032	1.632 ± 0.254	1.727 ± 0.540	1.289 ± 0.302
	**Day 5**	0.066 ± 0.058	0.556 ± 0.144	1.724 ± 0.469	1.969 ± 0.296	1.453 ± 0.333

**Figure 7:**
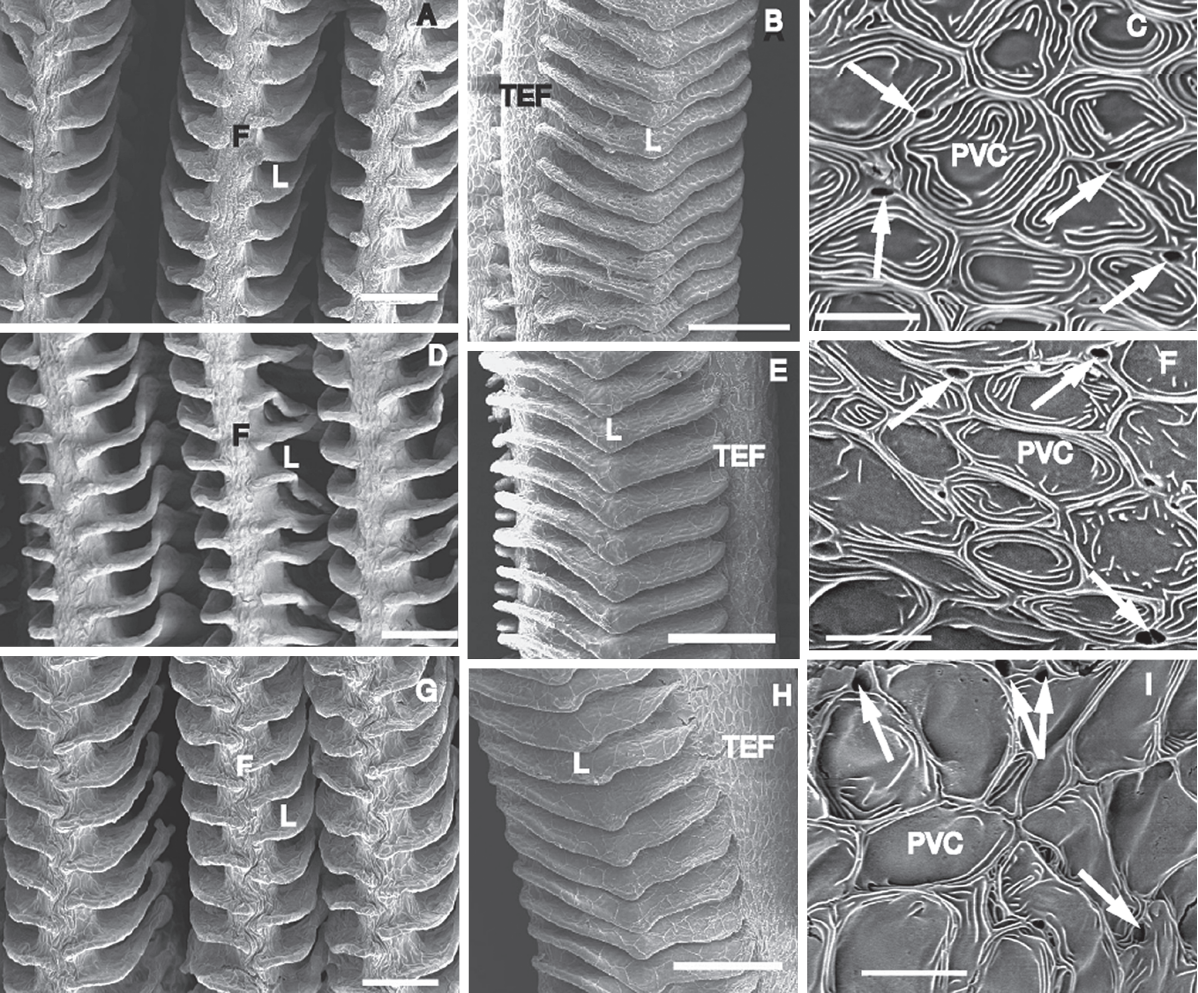
Scanning electron micrographs of gill filaments from Magadi tilapia before (**A**–**C**), and during 3 days (**D**–**F**) and 5 days of fasting (**G**–**I**). F—filament, L—lamella; PVC—pavement cell, TEF—trailing edge of filament. White arrows indicate the apical crypts of mitochondria-rich cells. Scale bars: A, D, G—50 μm; B, E, H—100 μm; C, F, I—10 μm. A: Frontal view of control gill filaments supporting long lamellae. B: Sagittal view of control gill filaments. Note roughly triangular shape of lamellae separated by large interlamellar spaces. C: Surface of control epithelium covering trailing edge of filament. Note pavement cells (PVC) ornamented with micro-ridges organized into a concentric pattern with a flat and smooth central area and small and deep apical crypts of mitochondria-rich cells (white arrow). D: Frontal view of 3-day fasted gill filaments. No difference compared to A. E: Sagittal view of 3-day fasted gill filaments. No difference compared to B. F: Surface of 3-day fasted filament epithelium. Note slightly expanded flat central area of PVC. Appearance of apical crypts of mitochondria-rich cells does not differ from that in C. G: Frontal view of 5-day fasted gill filaments. No difference compared to A and D. H: Sagittal view of 5-day fasted gill filaments. No difference compared to B and E. I: Surface of 5-day fasted filament epithelium. In PVCs, note highly expanded flat central area surrounding by few marginal micro-ridges. No difference is seen in appearance of apical crypts of mitochondria-rich cells compared to C and F.

Gross morphology of the gills did not show any changes over the fasting period, and there were no changes in lamellar dimensions and interlamellar cell mass at all (Fig. 7, Table 3). Although there were no differences in appearance and numbers of apical crypts of ionocytes, there was a clear difference in the appearance of the pavement cells. They showed micro-ridges organized into a concentric pattern with a flat and smooth central area in control fish, and this evolved to pavement cells with a highly expanded flat central area surrounded by few marginal micro-ridges in the fasted fish resulting in a reduced surface area (Fig. 7).

**Table 3:** Morphometric characteristics of gills from Magadi tilapia before (control) and during 3 days and 5 days of fasting; values are expressed as means and SEM (*n* = 4); no significant differences were detected

	Control	Experiment
	0 days	3 days	5 days
Basal length (L) of lamella, μm	95.4 ± 0.2	95.5 ± 0.3	95.5 ± 0.2
Height of ILCM, μm	12.4 ± 0.1	12.4 ± 0.2	12.5 ± 0.1
Distance (D) between lamellae, μm	21.7 ± 0.2	21.7 ± 0.2	21.8± 0.2
ILCM volume (V), μm^3^	2.56 × 10^5^	2.57 × 10^5^	2.57 × 10^5^

## Discussion

Due to the extremely demanding conditions they live in, Lake Magadi tilapia have some of the highest metabolic rates seen in fish. To sustain such high metabolic rates, a continuous energy supply is of vital importance (Wood *et al*., 1994). Recently, the lake has been subjected to siltation and flash floods due to human activities, which could endanger the growth of the cyanobacteria *Arthrospira* sp., the main food source of the tilapia. Therefore, our first aim was to determine if *A. grahami* would adjust their metabolic rates to a lower level when devoid of food. In previous studies at comparable temperatures, Lake Magadi tilapia showed their typical high routine metabolic rates with oxygen consumption rates between 14 and 21 μmol.g^−1^.h^−1^ (Narahara *et al*., 1996; Wood *et al*., 1994, 2002a, 2016). Our control values were in the lower range of these values, indicating that the fish had sufficiently recovered from capture. Surprisingly, and in contrast to our first hypothesis, no metabolic depression occurred, and instead, routine MO_2_ and swimming performance of *A. grahami* actually increased with 5 days of fasting indicating that aerobic metabolism was not compromised. In a previous study, attempting to measure the effects of fasting, a similar increase in routine metabolic rate was seen after 4 days (Wood *et al*., 1994). However, in that study, they could not exclude that the fish had fed on cyanobacteria growing on the walls of the holding tanks and on dead fish, which is why we took extreme care to avoid this by cleaning tanks daily, syphoning off any waste and removing any fish that were dead or dying three times a day. Clearly, the Lake Magadi tilapia do not economize their energy use to compensate for the lack of energy supply, instead maintaining a high metabolism, which could compromise their chances of survival when food becomes scarce.

Different species seem to respond to fasting in different ways. For example, in two closely related cyprinids, the goldfish *Carassius auratus* and the common carp *Cyprinus carpio*, goldfish sacrificed feeding metabolism to support swimming with similar MO_2_ and U_crit_ between fasted and fed fish (Liew *et al*., 2012), whereas in common carp, fasting reduced MO_2_ by approximately 35% when measured during routine swimming and aerobic exhaustion during a U_crit_ test (Liew *et al*., 2012; Shrivastava *et al*., 2017). Similarly, fasting juvenile rainbow trout (*Oncorhynchus mykiss*) and largemouth bass (*Micropterus salmoides*) showed a 30 and 41% lower MO_2_ respectively, compared to the control group (Alsop and Wood, 1997; Gingerich *et al*., 2010). In contrast, a study on another tropical cichlid, the oscar *A. ocellatus*, also found a steep increase in oxygen consumption rates with fasting (De Boeck *et al*., 2013) as was observed in the present study with *A. grahami*. In the oscars, this was mainly attributed to behavioural differences in the respirometers, with fed fish being much more passive compared to starved fish. However, due to the submerged amber respirometers in the present study, behavioural differences were not assessed. Interestingly, both the oscar and the Lake Magadi tilapia live in environments that are challenging for ion– and acid–base regulations. Oscars live in the Amazon basin, with warm, acidic and extremely soft, ion-poor water which can become hypoxic or even anoxic on the floodplains where these fish breed. In contrast, Lake Magadi tilapia live in even warmer, but extremely alkaline and ion-rich water, which can also become hypoxic and anoxic at night when the blue-green algae respire. Although the two environments are different, they both pose large demands on ion– and acid–base regulations, the cost of which for Lake Magadi tilapia has been estimated at up to 60% (Wood *et al*., 2002a). Therefore, metabolic depression might not be a valid option for these animals, and they vigorously look for food when deprived access to it.

Although metabolic depression did not occur, switches in fuel source were observed. There was a clear reduction in plasma lactate, due to reduced anaerobic metabolism and/or recycling of lactate to glucose to sustain increased use of carbohydrates. We cannot exclude that some of this reduction in lactate levels was due to attenuation of capture stress rather than cessation of feeding, but the increased use of carbohydrates seems to be supported by the accompanying reduction in muscle glycogen and a trend towards reduced plasma glucose levels. The reduced NQ, which is a function of nitrogen excretion divided by oxygen consumption rates, indicates that nitrogen-containing substrates become less important on a relative basis in the whole energy budget, however not on an absolute basis. In absolute numbers, urea-N excretion remained high at approximately 5 μmol-N.g^−1^.h^−1^ or 2.5 μmol.g^−1^.h^−1^ of urea. With a cost of 5 ATP per urea synthetized in the ornithine–urea cycle (Kirschner, 1993) and an estimated yield of 30 ATP per glucose using 6 O_2_ (or a P/O ratio of 2.5) in aerobic respiration, this means that 2.5 μmol.g^−1^.h^−1^ of O_2_ is used for urea synthesis or 18–13% of the measured aerobic respiration rates (14–19 μmol.g^−1^.h^−1^) of the Lake Magadi tilapia was designated towards urea production. Since nitrogen catabolism as such remained stable during the 5-day fasting period despite the increase in metabolic rate, it was now being fuelled by endogenous protein sources instead of the N-rich cyanobacteria as shown by the reduced protein levels in liver and muscle.

NQ was very high under control conditions and exceeded the already unusually high NQ of 0.20–0.27 from earlier measurements (Wood *et al*., 1994, 2002a). With a value of 0.35, it exceeded the maximal theoretical value for 100% protein oxidation *via* the citric acid cycle by deamination and amino acid oxidation under aerobic circumstances, which was estimated at 0.27 to 0.33 for fish tissue (Kutty, 1972; Kutty and Peer Mohamed, 1975; Kutty, 1978; Lyndon *et al*., 1992; De Boeck *et al*., 1995, 2000). However, glucogenic amino acids from protein breakdown such as alanine can also be converted to pyruvate and as such enter gluconeogenesis or be converted to lactate in anaerobic pathways. As indicated by the high plasma lactate levels, the high NQ suggests that the anaerobic component of metabolism in *A. grahami* was important. Such high plasma lactate levels have been observed before (Wood *et al*., 2013). However, after 5 days of fasting, when aerobic metabolism increased, lactate levels were reduced, and NQ dropped to 0.25 which is within the aerobic range. It is interesting to note that, in humans, Sola-Penna (2008) has suggested that lactate should not be regarded as simply an anaerobic metabolite but should be considered as a regulatory molecule that modulates the integration of metabolism and is therefore, together with metabolic acidosis, linked to protein catabolism.

**Figure 8:**
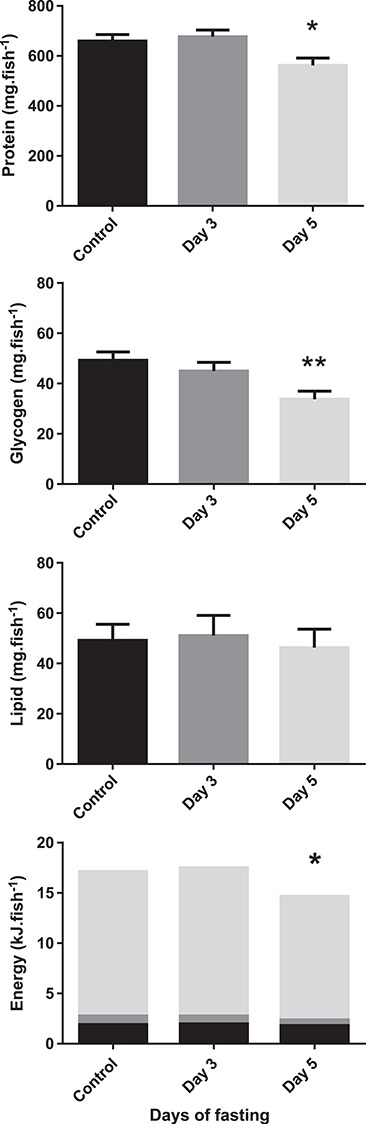
Whole-body energy stores (protein, glycogen and lipid) and energetic content (kJ) of Magadi tilapia before and during fasting (light grey: protein; dark grey: glycogen; black: lipids). Values are expressed as means and SEM. Asterisks indicate a significant difference between the fasted fish (*n* = 8) and the control fish (*n* = 8) (*P < 0.05, **P < 0.01)

At the average routine MO_2_ measured in our study (16.6 μmol/g/h), and assuming a linear increase over time, this metabolic rate corresponded to an average energy use of 0.9 kJ per day for a 5 g fish. When looking at the whole organism (Fig. 8) with the liver contributing about 1% to the total weight (Wood *et al*., 2002a and our own measurements), and muscle tissue estimated to contribute 50% of the total weight, protein was the most important energy source and contributed about 80% to the total available energy. From our measurements of liver and muscle energy stores, we calculated an energy loss of only 2.1 kJ. Assuming that during the first day, food was still available in the intestinal tract to sustain metabolism, this ‘budget’ lacks 1.5 kJ of consumed energy which cannot be explained by our data. A possible source could be visceral fat, which we did not assess. Of the 2.1-kJ energy drop that we did observe, protein represented 82%, followed by glycogen with a 13% energy loss and a non-significant loss of 5% for lipids. This is unusual, as fasting fish primarily utilize glycogen as an immediate energy source, followed by lipid stores and only then is protein mobilized (Alliot *et al*., 1984; Echevarría *et al*., 1997; Pastoureaud, 1991). A previous study on common carp and goldfish using a 7-day fasting period also showed that the former primarily relied on liver and muscle glycogen, while the latter mainly used protein (Liew *et al*., 2012). In that study, protein stores were also reduced in common carp, although to a lesser extent, and lipid stores remained stable. Also, others have suggested that protein is the main fuel used by fish (Van Den Thillart, 1986; Jobling, 1994).

Lake Magadi tilapia are different in their nitrogen metabolism compared to other freshwater teleosts, being a 100% ureotelic. Plasma urea concentration and urea-N excretion were not affected by the 5-day deprivation of the N-rich food source, a finding that supports rejection of our second hypothesis that a reduction in urea-N excretion would be seen when nitrogen input ceased. Urea transporters were not directly quantified by Western blot or immunohistochemistry but expression of branchial and intestinal urea transporter mRNA was only transiently downregulated at Day 3, and they were back to control levels at Day 5. So nitrogen/protein metabolism remained important and seems to be hardwired, as is also seen in ureotelic elasmobranchs (Kajimura *et al*., 2008), even though some extra energy was supplied by carbohydrates. Notably, similar hardwiring (continued production of urea-N and no ammonia) was seen when Magadi tilapia were adapted to circumneutral fresh water, where excretion of ammonia would become possible (Wood *et al*., 2002a). However, the gene expression of the ammonia transporting Rh proteins and glutamine synthetase were reduced in the gills. Despite their ureotelic excretion mechanisms, the high metabolic rates and nitrogen dependence of Lake Magadi tilapia likely produce large amounts of ammonia internally, necessitating cellular ammonia handling. Tissue ammonia levels are comparable to those of other teleosts, and the presence of Rh protein in Magadi tilapia gill tissue has been confirmed both by gene expression studies and by immunohistochemistry (Wood *et al*., 2013). Expression of Rh glycoprotein mRNA responds to ammonia exposure confirming their role in ammonia transport in these fish (Wood *et al*., 2013). Glutamine synthetase is especially involved in N-sequestration and amino acid synthesis rather than protein catabolism, or in detoxifying ammonia. The simultaneous reduction in gene expression in both glutamine synthetase and Rh protein suggests that plasma and/or tissue ammonia levels might have been reduced during fasting. Regrettably, our attempts to measure plasma ammonia failed, and ammonia metabolism in these fish is an area of research that certainly warrants further research. On a final note, Rh glycoproteins have also been suggested to facilitate CO_2_ transport (Perry *et al*., 2010) and in mammals even HCO_3_^−^ transport (Huang and Ye, 2010) which is interesting because the Magadi tilapia must actively excrete HCO_3_^−^ across their gills (Wood *et al*., 2012), and plasma total CO_2_ concentration (or [HCO_3_^−^]) was decreased during fasting.

In previous studies, urea-N excretion did not increase with increasing MO_2_ when Magadi tilapia were subjected to either exhaustive exercise (Wood *et al*., 1989) or aerobic steady-state exercise (Wood *et al*., 2016). It was suggested that this could have been due to an inhibition of ureagenesis resulting from low HCO_3_^−^ availability during metabolic acidosis (Wood *et al*., 1994), or to an inhibition of urea synthesis to reduce costs during aerobic exercise (Wood *et al*., 2016). Our data also indicate that these fish depend on a stable urea-N excretion rate. They support the view by Wood *et al*. (1994, 2002a) that urea-N excretion is not linked to acid–base regulation and is regulated independently. This was evidenced by plasma total CO_2_ concentration (i.e. [HCO_3_^−^]) which decreased substantially after fasting independent of the stable urea excretion rates.

Together with the reduced plasma total CO_2_, we observed reductions in plasma Na^+^ and osmolality, but not in plasma Cl^−^. This is surprising, as net transepithelial electrochemical gradients at the gills drive Na^+^ and HCO_3_^−^ inwards while Cl^−^ experiences a small outward pressure, at least in recently fed fish (Wood *et al*., 1994, 2012). Normally, this entry of Na^+^ and basic equivalents occurs either via passive diffusion across the gills or as a result of drinking. Indeed, Lake Magadi tilapia resemble marine fishes in many aspects. They show marine-type ionocytes including accessory cells (Maina, 1990; Laurent *et al*., 1995; Wood *et al*., 2002b) and imbibe the surrounding water at rates of about 8 mL.kg^−1^.h^−1^ (Maloiy *et al*., 1978; Wood *et al*. 2002b). When they are not feeding, i.e. at night, the stomach is bypassed and contains very little water (Bergman *et al*., 2003). Absolute differences in drinking rates were not reported in this study, but it is possible that drinking and/or absorption rates were lower in the fish with empty stomachs. This would greatly reduce the ion- and base-load that the fish experienced and could explain the lowered plasma Na^+^ and total CO_2_ levels. A simultaneous reduction in Na^+^ and HCO_3_^−^ without change in Cl^−^ is consistent with metabolic acidosis, in accord with strong ion difference theory Stewart (1983), ensuring electroneutrality in the plasma.

Although respiration rates were high and urea-N excretion remained stable, we cannot exclude reduced inwards diffusion of Na^+^ and base at the gills. In control fish, PVCs were ornamented with micro-ridges organized into a concentric pattern with a flat and smooth central area as described before for PVCs located on the filament (Maina, 1990; Laurent *et al*., 1995; Wood *et al*., 2002b). In fasted fish, PVCs showed a highly expanded flat central area surrounding by a few marginal micro-ridges. This would reduce the exchange surface area for diffusion of base and Na^+^ into the fish. If this reduction in micro-ridges on the PVC surface would be caused by swelling of the PVC as a consequence of the lowered osmolality in the plasma, diffusion distance would be increased as well, lowering diffusion even further. Ionocytes that are linked to base excretion rather than uptake (Laurent *et al*., 1995) remained unchanged.

In conclusion, *A. grahami* responded remarkably well to a 5-day fasting period, which is reassuring from a conservation point of view as it shows that they have at least some resilience towards short periods of food deprivation. In contrast to our original hypothesis, aerobic metabolism and performance went up, and urea-N excretion remained stable. Protein remained the main energy source, now originating from proteolysis in both liver and muscle, which was supplemented by muscle glycogenolysis. In contrast to these short-term effects, the sustained high metabolism and preference for proteolysis are worrying in case longer periods of low food availability would occur. The apparent inability of Magadi tilapia to lower their metabolic rates and nitrogen metabolism necessitate a continuous energy supply in the long run. At the moment, possible mitigating measures that are being considered to avoid siltation and flash floods include practicing safe farming by constructing terraces, the diversions of rivers, building of check dams in order to ease velocity of water, and the construction of water catchment areas upstream (Ministry of Environment and Forestry, 2015). Even though these efforts are driven by the need to keep the soda crust devoid of impurities so as to allow its continued commercial exploitation, they would also protect the exceptional Lake Magadi tilapia. Other threats however remain, extracting water from the lagoons and aquifers through boreholes will influence the size, and water composition of these peripheral pools as the evaporating water will not be replenished sufficiently. Therefore, a more integrated water management plan is essential.

## References

[ref1] Geographic A (2011) A fish apart. Africa Geographic May 2011: 20.

[ref2] Alliot E, Djabali M, Pastoureaud A, Thebault H (1984) Changes in the biochemical composition of tissues in juvenile sea bass during forced starvation. Biochem Syst Ecol 12: 200–213.

[ref3] Alsop DH, Wood CM (1997) The interactive effects of feeding and exercise on oxygen consumption, swimming performance and protein usage in juvenile rainbow trout (*Oncorhychus mykiss*). J Exp Biol 200: 2337–2346. 9320259 10.1242/jeb.200.17.2337

[ref4] Atkinson DE (1992) Functional roles of urea synthesis in vertebrates. Physiol Zool 65: 243–267.

[ref5] Bayona J, Akinyi E (2006) *Alcolapia grahami* In The IUCN Red List of Threatened Species 2006: e.T60453A12368415.

[ref6] Bergman AN, Laurent P, Otiang’a-Owiti G, Bergman HL, Walsh PJ, Wilson P, Wood CM (2003) Physiological adaptations of the gut in the Lake Magadi tilapia, *Alcolapia grahami*, an alkaline- and saline-adapted fish. Comp Biochem Physiol 136A: 701–715. 10.1016/s1095-6433(03)00223-x 14613798

[ref7] Bligh EG, Dyer WJ (1959) A rapid method of total lipid extraction and purification. Can J Biochem Physiol 37: 911–917. 13671378 10.1139/o59-099

[ref8] Bradford MM (1976) A rapid and sensitive method for the quantitation of micro-gram quantities of protein utilizing the principle of protein–dye binding. Anal Biochem 72: 249–254. 10.1016/0003-2697(76)90527-3 942051

[ref9] Brett JR (1964) The respiratory metabolism and swimming performance of young sockeye salmon. J Fish Res Board Can 21:1183–1226.

[ref10] Brown JA, Watson J, Bourhill A, Wall T (2008) Evaluation and use of the lactate pro, a portable lactate meter, in monitoring the physiological well-being of farmed Atlantic cod (Gadus morhua). *Aquaculture* 285: 135–140.Coe MJ (1966) the biology of *Tilapia grahami* Boulenger in Lake Magadi, Kenya. Acta Trop 23: 146–177.

[ref11] Cooke SJ et al. (2008) Effects of different capture techniques on the physiological condition of bonefish Albula vulpes evaluated using field diagnostic tools. J Fish Biol 73: 1351–1375.

[ref12] De Boeck G, De Smet H, Blust R (1995) The effect of sublethal levels of copper on oxygen consumption and ammonia excretion in common carp, *Cyprinus carpio*. Aquat Toxicol 32: 127–141.

[ref13] De Boeck G, Vlaeminck A, van der Linden A, Blust R (2000) The energy metabolism of common carp (*Cyprinus carpio*) when exposed to salt stress: an increase in energy expenditure or effects of starvation? Physiol Biochem Zool 73: 102–111. 10685912 10.1086/316717

[ref14] De Boeck G, Wood CM, Iftikar FI, Matey V, Scott GR, Sloman KA, da Silva MNP, Almeida-Val VMF, Val AL (2013) Interactions between hypoxia tolerance and food deprivation in Amazonian Oscars, *Astronotus ocellatus*. J Exp Biol 216: 4590–4600. 24072802 10.1242/jeb.082891

[ref15] Echevarría G, Martínez-Bebiá M, Zamora S (1997) Evolution of biometric indices and plasma metabolites during prolonged starvation in European sea bass (*Dicentrarchus labrax*, L.). Comp Biochem Physiol A118: 111–123.

[ref16] Franklin CE, Crockford T, Johnston IA, Camunde C (1995) Scaling of oxygen consumption in Lake Magadi tilapia, *Oreochromis alcalicus grahami*: a fish living at 37°C. J Fish Biol 46: 829–834.

[ref17] Gingerich AJ, Philipp DP, Suski CD (2010) Effects of nutritional status on metabolic rate, exercise and recovery in a freshwater fish. J Comp Physiol B 180: 371–384. 19936760 10.1007/s00360-009-0419-4

[ref18] Huang C-H, Ye M (2010) The Rh protein family: gene evolution, membrane biology, and disease association. Cell Mol Life Sci 67: 1203–1218. 19953292 10.1007/s00018-009-0217-x PMC11115862

[ref19] Jobling M (1994) Fish Bioenergetics. Chapman & Hall, New York

[ref20] Johansen K, Maloiy GMO, Lykkeboe G (1975) A fish in extreme alkalinity. Resp Physiol 24: 156–162. 10.1016/0034-5687(75)90110-3 241103

[ref21] Johannsson OE et al. (2014) Air breathing in the Lake Magadi tilapia *Alcolapia grahami*, under normoxic and hyperoxic conditions, and the association with sunlight and ROS. J Fish Biol 84: 844–863. 24673646 10.1111/jfb.12289

[ref22] Kajimura M, Walsh PJ, Wood CM (2008) The spiny dogfish *Squalus acanthias* L. maintains osmolyte balance during long-term starvation. J Fish Biol 72: 656–670.

[ref23] Karnovsky MJ (1965) A formaldehyde-glutaraldehyde fixative of high osmolality for use in electron microscopy. J Cell Biol 27: A137.

[ref24] Kavembe GD, Meyer A, Wood CM (2016) Fish populations in east African saline lakes In Schagerl M, ed, Soda Lakes of East Africa. Springer International Publishing, Ed, Switserland, pp. 227–257

[ref25] Channel 1 KBC (2015) #ECOWATCH: focus on Lake Magadi degradation Posted 15 July 2015, https://www.youtube.com/watch?v=P5taOWFJmNo

[ref26] Kirschner LB (1993) The energetics of osmotic regulation in ureotelic and hypoosmotic fishes. J Exp Zool 267: 19–26.

[ref27] Kutty MN (1972) Respiratory quotients and ammonia excretion in *Tilapia mossambica*. Mar Biol 16: 126–133.

[ref28] Kutty MN (1978) Ammonia quotient m sockeye salmon (*Oncorhynchus nerka*). J Fish Res Board Can 35: 1003–1005.

[ref29] Kutty MN, Peer Mohamed M (1975) Metabolic adaptations of mullet *Rhinomugil corsula* (Hamilton) with special reference to energy utilisation. Aquaculture 5: 253–270.

[ref30] Laurent P, Maina JN, Bergman HL, Narahara A, Walsh PJ, Wood CM (1995) Gill structure of a fish from an alkaline lake - effect of short-term exposure to neutral conditions. Can J Zool 73: 1170–1181.

[ref31] Liew HJ, Sinha AK, Mauro N, Diricx M, Blust R, De Boeck G (2012) Fasting goldfish, *Carassius auratus*, and common carp, *Cyprinus carpio*, use different metabolic strategies when swimming. Comp Biochem Physiol A 163: 327–335. 10.1016/j.cbpa.2012.07.012 22884681

[ref32] Lindley TE, Schreiderer CL, Walsh PJ, Wood CM, Bergman HL, Bergman AL, Laurent P, Wilson P, Anderson PM (1999) Muscle as the primary site of urea cycle enzyme activity in an alkaline lake-adapted tilapia, *Oreochromis alcalicus grahami*. J Biol Chem 274: 29858–29861. 10514466 10.1074/jbc.274.42.29858

[ref33] Lyndon AR, Houlihan DF, Hall SJ (1992) The effect of short-term fasting and a single meal on protein synthesis and oxygen consumption in cod, *Gadus morhua*. J Comp Physiol B 162: 209–215. 1377211 10.1007/BF00357525

[ref35] Maina JN (1990) A study of the morphology of the gills of an extreme alkalinity and hyperosmotic adapted teleost *Oreochromis alcalicus grahami* (Boulenger) with particular emphasis on the ultrstructure of the chloride cells and their modification with water dilution. A SEM and TEM study. Anat Embryol 181: 83–98. 2305972 10.1007/BF00189731

[ref37] Maloiy GMO, Lykkeboe G, Johansen K, Bamford OS (1978) Osmoregulation in *Tilapia grahami*: a fish in extreme alkalinity In Schmidt-Nielsen K, Bolis L, Maddrell SHP, eds, Comparative physiology: water, ions and fluid mechanics. Cambridge University Press, Cambridge, pp. 229–238

[ref38] Meijer AJ, Lamers WH, Chamuleau FM (1990) Nitrogen metabolism and ornithine cycle function. Physiol Rev 70: 701–748. 2194222 10.1152/physrev.1990.70.3.701

[ref40] Ministry of Environment and Forestry (2015) Human activities upstream hurting Lake Magadi. http://www.environment.go.ke/?p=2517.

[ref41] Narahara A, Bergman HL, Laurent P, Maina JN, Walsh PJ, Wood CM (1996) Respiratory physiology of Lake Magadi tilapia (*Oreochromis alcalicus grahami*), a fish adapted to a hot, alkaline, and frequently hypoxic environment. Physiol Zool 69: 1114–1136.

[ref42] Pastoureaud A (1991) Influence of starvation at low temperatures on utilization of energy reserves, appetite recovery and growth character in sea bass, *Dicentrarchus labrax*. Aquaculture 99: 167–178.

[ref43] People Daily (2018) Siltation chokes life out of Lake Magadi Posted July 27, 2018, http://www.mediamaxnetwork.co.ke/news/siltation-chokes-life-out-of-lake-magadi-454554/

[ref44] Perry SF, Braun MH, Noland M, Dawdy J, Walsh PJ (2010) Do zebrafish Rh proteins act as dual ammonia-CO_2_ channels? J Exp Zool A 313: 618–621. 10.1002/jez.631 20683854

[ref45] Price NM, Harrison PJ (1987) Comparison of methods for the analysis of urea in seawater. Mar Biol 94: 307–313.

[ref46] Randall DJ, Wood CM, Perry SF, Bergman HL, Maloiy GMO, Mommsen TP, Wright PA (1989) Ureotelism in a completely aquatic teleost fish: a strategy for survival in an extremely alkaline environment. Nature 337: 165–166. 2911349 10.1038/337165a0

[ref47] Randall DJ, Burggren W, French K (1997) Using energy: meeting environmental challenges In Eckert Animal Physiology: mechanisms and adaptations, Ed4th WH Freeman and Company, pp. 665–723.

[ref48] Roe JH, Dailey RE (1966) Determination of glycogen with the anthrone reagent. Anal Biochem 15: 245–250. 4289896 10.1016/0003-2697(66)90028-5

[ref49] Serra-Llinares RM, Tveiten H, Damsgård B, Aas-Hansen O (2012) Evaluation of a fast and simple method for measuring plasma lactate levels in Atlantic cod, *Gadus morhua* (L.). Intern J Fish Aquacult 4: 217–220.

[ref50] Shrivastava J, Sinha AK, Cannaerts S, Blust R, De Boeck G (2017) Temporal assessment of metabolic rate, ammonia dynamics and ion-status in common carp during fasting: a promising approach for optimizing fasting episode prior to fish transportation. Aquaculture 481: 218–228.

[ref51] Sola-Penna M (2008) Metabolic regulation by lactate. IUBMB Life 60: 605–608. 18506840 10.1002/iub.97

[ref52] Sollid J, De Angelis P, Gundersen K, Nilsson GE (2003) Hypoxia induces adaptive and reversible gross morphological changes in crucian carp gills. J Exp Biol 206: 3667–3673. 12966058 10.1242/jeb.00594

[ref53] Stewart PA (1983) Modern quantitative acid–base chemistry. Can J Physiol Pharmacol 61: 1444–1461. 6423247 10.1139/y83-207

[ref54] Stoot LJ, Cairns NA, Cull F, Taylor JJ, Jeffrey JD, Morin F, Mandelman JW, Clark TD, Cooke SJ (2014) Use of portable blood physiology point-of-care devices for basic and applied research on vertebrates: a review. Conserv Physiol 2. doi: 10.1093/conphys/cou011. PMC4806731 27293632

[ref55] Van Den Thillart G (1986) Energy metabolism of swimming trout (*Salmo gairdneri*). Comp Biochem Physiol B 156: 511–520.

[ref56] Wood CM, Perry SF, Wright PA, Bergman HL, Randall DJ (1989) Ammonia and urea dynamics in the Lake Magadi tlapia, a ureotelic teleost fish adapted to an extremely alkaline environment. Resp Physiol 77: 1–20. 10.1016/0034-5687(89)90025-x 2799103

[ref57] Wells RMG, Pankhurst NW (1999) Evaluation of simple instruments for the instruments for the measurement of blood glucose and lactate, and plasma protein as stress indicators in fish. J World Aquacult Soc 30: 276–284.

[ref58] Wood CM, Bergman HL, Laurent P, Maina JN, Narahara A, Walsh PJ (1994) Urea production, acid-base regulation and their interactions in the Lake Magadi tilapia, a unique teleost adapted to a highly alkaline environment. J Exp Biol 189: 13–36. 9317245 10.1242/jeb.189.1.13

[ref59] Wood CM, Wilson P, Bergman HL, Bergman AN, Laurent P, Otiang’a-Owiti G, Walsh PJ (2002a) Obligatory urea production and the cost of living in the Magadi tilapia revealed by acclimation to reduced salinity and alkalinity. Physiol Biochem Zool 75: 111–122. 12024287 10.1086/340626

[ref60] Wood CM, Wilson P, Bergman HL, Bergman AN, Laurent P, Otiang’a-Owiti G, Walsh PJ (2002b) Ionoregulatory strategies and the role of urea in the Magadi tilapia (*Alcolapia grahami*). Can J Zool 80: 503–515.

[ref61] Wood CM et al. (2012) Transepithelial potential in the Magadi tilapia, a fish living in extreme alkalinity. J Comp Physiol B 182: 247–258. 21912898 10.1007/s00360-011-0614-y

[ref62] Wood CM et al. (2013) Rh proteins and NH_4_^+^-activated Na^+^-ATPase in the Magadi tilapia (*Alcolapia grahami*), a 100% ureotelic teleost fish. J Exp Biol 216: 2998–3007. 23885087 10.1242/jeb.078634

[ref63] Wood CM et al. (2016) Mammalian metabolic rates in the hottest fish on earth. Scientific Reports 6: 26990. 27257105 10.1038/srep26990 PMC4891707

